# Survival, mortality and epidemic risk status of COVID-19: a population-based Study in Golestan province, Iran

**DOI:** 10.1186/s13690-024-01330-4

**Published:** 2024-07-08

**Authors:** Fatemeh Kashiri, Parvin Sarbakhsh, Asghar Mohammadpoorasl, Navisa Sadat Seyedghasemi, Ali Bagheri, Hossein Akbari

**Affiliations:** 1 Department of Statistics and Epidemiology, Faculty of Health, Tabriz University of Medical Sciences, Tabriz, Iran; 2 Department of Biostatistics and Epidemiology, Faculty of Health, Golestan University of Medical Sciences, Gorgan, Iran; 3 Communicable Disease Control of Health Center, Golestan University of Medical Sciences, Gorgan, Iran

**Keywords:** COVID-19, Mortality, Hospital Mortality, Survival analysis, Pandemics, Pandemic preparedness

## Abstract

**Background:**

Appreciating the various dimensions of the coronavirus disease 2019 (COVID-19) pandemic can improve health systems and prepare them to deal better with future pandemics and public health events. This study was conducted to investigate the association between the survival of hospitalized patients with COVID-19 and the epidemic risk stratification of the disease in Golestan province, Iran.

**Methods:**

In this study, all patients with COVID-19 who were hospitalized in the hospitals of Golestan province of Iran from February 20, 2020, to December 19, 2022, and were registered in the Medical Care Monitoring Center (MCMC) system (85,885 individuals) were examined.The community's epidemic risk status (ERS) was determined based on the daily incidence statistics of COVID-19. The survival distribution and compare Survival in different subgroups was investigated using Kaplan–Meier and log-rank test and association between the survival and ERS by multiple Cox regression modeling.

**Results:**

Out of 68,983 individuals whose data were correctly recorded, the mean age was 49 (SD = 23.98) years, and 52.8% were women. In total, 11.1% eventually died. The length of hospital stay was varying significantly with age, gender, ERS, underlying diseases, and COVID-19 severity (*P* < 0.001 for all). The adjusted hazard ratio of death for the ERS at medium, high, and very high-risk status compared to the low-risk status increased by 19%, 26%, and 56%, respectively (*P* < 0.001 for all).

**Conclusions:**

Enhancing preparedness, facilitating rapid rises in hospital capacities, and developing backup healthcare capacities can prevent excessive hospital referrals during health crises and further deaths.

**Table:** 

Text box 1. Contributions to the literature
• **Lessions learned of COVID-19 should be used to increase health system preparedness for the next pandemics and public health events.**
• **Increment in the Epidemic Risk Status (ERS) levels (obtaibed from COVID-19 risk assement tool of ministry of health of Iran) increase the risk of death in hospitalized patients with COVID-19.**
• **Implementing community-level measures to mitigate COVID-19 transmission can enhance the resilience of the health system by reducing excessive patient referrals and hospital overburdenment.**
• **Strengthening surge capacities and preparing health care facilities can mitigate the potential of overwhelming health system and as a result reducing mortality.**

## Introduction

The coronavirus disease 2019 (COVID-19) pandemic caused more than 6.9 million deaths worldwide between December 2019 and December 2023 [1, 2]. Besides significantly damaging multiple dimensions of human life, the pandemic placed an immense financial burden on health systems and triggered equipment shortages [3–5]. Iran was the first country in the Middle East and one of the first countries in the world where COVID-19 was reported, with the Golestan province representing one of the first and most severely affected regions [6, 7].

The most critical challenge for health and treatment systems in dealing with COVID-19 has been to reduce the incidence and mortality of this disease. Therefore, understanding the factors affecting the death of patients with this disease is of great interest. Based on previous studies, individual risk factors such as old age and underlying diseases (e.g., diabetes, cardiovascular diseases, cancer, hypertension, or compromised immunity) have a proven role in disease exacerbation, hospitalization, and mortality of patients with COVID-19 [8, 9]. Also, some studies have shown that with an upsurge in hospital admissions and delay in the time of referral to the hospital, the case fatality rate (CFR) increases [10–13]. In addition, evidence shows that the CFR of hospitalized COVID-19 patients differed in different waves of the pandemic [14]. An excessive increase in referrals to the treatment system and a high ratio of hospital bed occupancy, which generally occurs during peaks of an epidemic, may reduce the quality of services provided to hospitalized patients, trigger delays in service provision, and alter admission and discharge procedures to prioritize the allocation of hospital beds and critical care resources [15]. However, there is still limited evidence on changes in COVID-19 deaths and the impact of different epidemic risk status (ERS) levels on the CFR of patients, especially in Iran.

It is obvious that this is not the last epidemic, and it is necessary for healthcare systems to use the lessons learned from COVID-19 as much as possible to improve their preparedness and ability to respond to pandemics and epidemics. Therefore, in the present study, we investigated the changes in CFR in patients hospitalized with COVID-19 in the hospitals of Golestan province, attempting to diverge the relationship between the ERS and the survival of these patients by taking into account the influence of risk factors like age, gender, underlying diseases, and COVID-19 severity.

## Methods

### Study design and participants

This retrospective study included 85,885 patients registered in the national Medical Care Monitoring Center (MCMC) system and admitted from 02/20/2020 to 12/19/2022 to hospitals affiliated with Golestan University of Medical Sciences, Iran.

The MCMC system is run by Iran’s Ministry of Health and Medical Education, operating in all medical science universities as the most complete system for registering outpatient and inpatient cases of COVID-19. This system records patients' information, including personal characteristics such as age, gender, city of residence, being a treatment staff, history of underlying diseases, signs and symptoms, disease outcome, and the date of occurrence. Patients registered in this system include definite cases (with a positive polymerase chain reaction test or positive lung computed tomography scan) or probable cases of COVID-19 (diagnosed clinically by a physician).

**Fig. 1:**
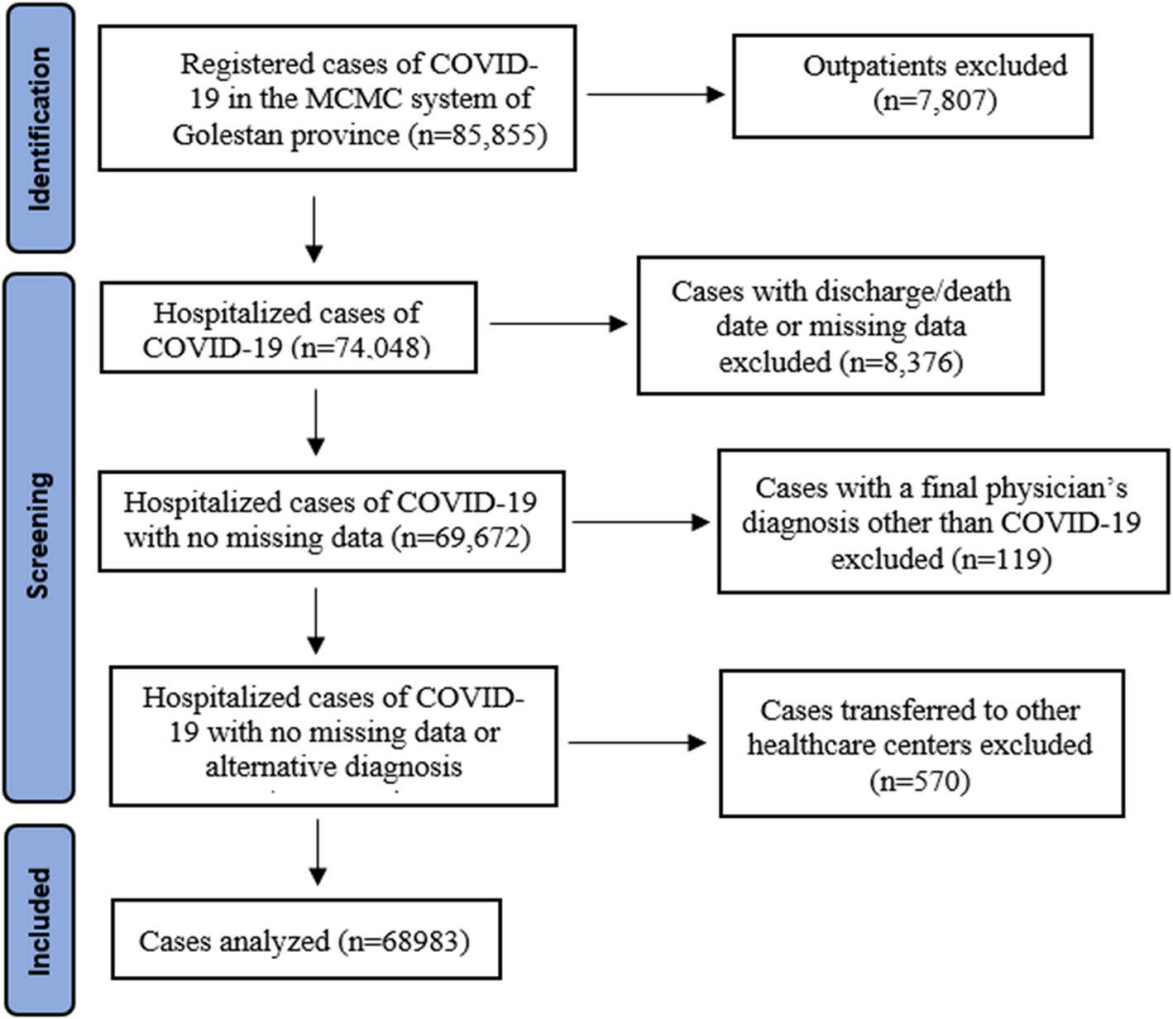
Flowchart of the process of sampling hospitalized patients with COVID-19 in the Golestan province

After extracting the data in the investigated period (85,855 people), we refined the data to remove outpatients. We also excluded hospitalized patients who had no date of discharge/death, had missing individual data, or had been transferred to other medical centers excluded from the coverage of Golestan University of Medical Sciences. Finally, the data of 68,983 people were analyzed (Fig. 1).

### Patient survival time

Based on the date of admission, the date of death (failure) or discharge from the hospital (censor) was calculated in terms of days. In this study, the survival time was measured and recorded as the length of hospitalization of patients in the hospital, i.e., the time from the start of hospitalization to the time of discharge (as the length of hospitalization) or the time of death.

### Epidemic risk status (ERS)

The official risk assessment method announced by the Ministry of Health and the National COVID-19 Management Committee was used to determine the ERS. This method classifies the epidemic into four ERS levels based on the adjusted incidence rate (AIR):

$$AIR=\frac{{N}_{P} +{ \alpha }( {N}_{N}- {N}_{U}) }{\text{Pop }*\text{ t}}*\text{100,000}$$

- ${N}_{P}$: The number of hospitalized severe acute respiratory illness (SARI) cases with positive COVID-19 test results.
- ${N}_{N}$: Number of hospitalized SARI cases whose COVID-19 test results are negative.
- ${N}_{U}$: Number of hospitalized SARI cases whose COVID-19 test results have not been recorded.
- $$\text{Pop}$$: City population
- t: The length of the target time period in terms of days.
- α: The impact coefficient of negative or uncertain cases. This coefficient is considered due to the false negative probability of the molecular test.

The following formula was used to calculate the risk score based on the AIR:

$$Score={\text{AIRw}}_{1}+\updelta *\text{Max }( 0, {\text{AIRw}}_{1}-\frac{{\text{AIRw}}_{2} +{\text{AIRw}}_{3}}{2})$$

- ${\text{AIRw}}_{1}$: Adjusted incidence rate for the last week.
- ${\text{AIRw}}_{2}$: Adjusted incidence rate for the week prior to the last week.
- ${\text{AIRw}}_{3}$: Adjusted incidence rate for the week two weeks prior to the last week.

In the above formula, the AIR increase in the last week compared to the previous 14-day period is calculated to determine the increasing or decreasing trend of the disease’s incidence. If the trend is decreasing or constant, only ${\text{AIRw}}_{1}$ is used. If the trend is increasing, the increment is added to ${\text{AIRw}}_{1}$ by taking into account the delta coefficient.

Finally, based on the ERS index, cities are classified according to the following criteria:

- **Very high risk (red):** ERS score > 4 AND increasing trend
- **High risk (orange):** ERS score 2–4 or ERS score > 4 AND decreasing trend
- **Moderate risk (yellow):** ERS score of 1–2
- **Low risk (blue):** ERS score < 1

For each of the registerd patients, ERS level in the first day of hospital admission is considered as the base of comparisons.

### Other variables (covariates)

Based on the age classification of COVID-19 deaths recorded by the Centers for Disease Control and Prevention (CDC), the age of the individuals was classified into eight groups: 0–17 years, 18–29 years, 30–39 years, 40–49 years, 50–64 years, 65–74 years, 75–84 years, and ≥ 85 years [16]. Individuals were classified as having an underlying disease if they had at least one of the following: heart disease, diabetes, chronic kidney disease, asthma or other chronic lung diseases, chronic neurological disorders, hypertension, blood disorder, chronic liver disease, cancer, HIV/AIDS, acquired or congenital immunodeficiency, other chronic diseases, or pregnancy. Otherwise, the person was classified as having no underlying diseases.

The clinical spectrum of COVID-19 was classified based on the American National Institutes of Health’s Guidelines for the Treatment of COVID-19 [17]:

- Moderate illness: Patients with clinical or imaging evidence of lower respiratory disease and an arterial oxygen level > 94%.
- Severe illness: People with arterial oxygen level < 94%, respiratory rate > 30 times per minute, opacities covering > 50% of the lungs on imaging, and requiring oxygen therapy.
- Critical illness: People hospitalized in the intensive care unit or intubated (respiratory failure, septic shock, or multiple organ failure).

### Statistical analysis

Findings are reported using frequencies and percentages for all variables. The overall survival rate was evaluated using the Kaplan–Meier method, and the assumption of proportional hazards based on the Schoenfeld scale. Also, the comparison of survival of patients in different subgroups and the correlation of ERS with death/survival was assessed using the log-rank test and univariate and multivariate Cox modeling. All analyses were performed using SPSS 26 and Stata 17 software.

## Results

Out of a total of 68,983 people (80.34% of the primary data) subjected to the final analysis, 32,534 people (47.2%) were men, and 36,449 people (52.8%) were women. The mean (SD) age of these hospitalized patients was 49 (23.98) years and total of 29,705 people (43.1%) had at least one underlying disease (Table 1). The most common underlying diseases were hypertension (19.8%), diabetes (15.5%), cardiovascular disease (13.2%), asthma (4.0%) and chronic kidney diseases (2.1%) respectively.

**Table 1:** Baseline charachteristics and death rate of COVID-19 hospitalized patients in Golestan province, Iran

Variables	Classification	Outcome			
		Death	Discharge	Total	
		Frequency (%)	Frequency (%)	Number	*P*-value*
Age (years)	0-17	259 (2.9)	8684 (97.1)	8943	
	18-29	132 (3)	4257 (97)	4389	
	30-39	316 (3.9)	7736 (96.1)	8052	
	40-49	657 (7.3)	8386 (92.7)	9043	
	50-64	2173 (12.2)	15577(87.8)	17750	<0.001^a^
	65-74	1978 (17.4)	9372 (82.6)	11350	
	75-84	1443 (21.7)	5212 (78.3)	6655	
	<85	725 (25.9)	2076 (74.1)	2801	
Gender	Women	3740 (10.3)	32709 (89.7)	36449	<0.001^a^
	Men	3943 (12.1)	2859 (87.9)	32534	
Treatment staff	Yes	10 (4.4)	217 (95.6)	227	0.002^a^
	No	6051 (10.8)	49776 (89.2)	55827	
Pregnancy	Yes	5 (.3)	1865 (99.7)	1870	<0.001^a^
	No	3835 (10.8)	30844 (89.2)	34579	
Underlying disease	Yes	3614 (12.2)	26091 (87.8)	29705	<0.001^a^
	No	4069 (10.4)	35209 (89.6)	39278	

^*^ chi-square test

The number of people with the clinical spectrum of moderate, severe, and critical COVID-19 was 33,975 (49.2%), 25,671 (37.2%), and 9,355(13.6%), respectively. The most common symptoms were muscle pain (78.3%), cough (44.4%), respiratory distress (43.2%), and fever (42.6%). The distribution of symptoms and signs of hospitalized patients is presented in Table 2.

**Table 2:** Clinical and paraclinical charchteristics of COVID-19 hospitalized patients in Golestan province, Iran

Clinical or preclinical characteristics		Outcome		
		Death (*n*=7683)	Discharge (*n*=61300)	Total (*n*=68983)
		Frequency (%)	Frequency (%)	Frequency (%)
Fever		2429 (31.6)	26926 (43.9)	29355(42.6)
Cough		2645 (34.4)	27966 (45.6)	30611(44.4)
Muscle pain		1218 (15.9)	13748 (22.4)	14966(21.7)
Respiratory distress		4560 (59.4)	25220 (41.1)	29780(43.2)
Loss of consciousness		1763 (22.9)	2462 (4.0)	4225(6.1)
Loss/ reduction sense of smell		34 (0.4)	535 (0.9)	569(0.8)
Loss/reduction of sense of taste		22 (0.3)	449 (0.7)	471(0.7)
convulsions		67 (0.9)	712 (1.2)	779(1.1)
Headache		320 (4.2)	5752(9.5)	6072(8.9)
Dizziness		174 (2.3)	2119 (3.5)	2293(3.4)
Chest pain		196(2.6)	2490(4.1)	2686(4.0)
paresis of organs		39(0.5)	236 (0.4)	275(0.4)
Limb plegia		35(0.5)	182 (0.3)	217(0.3)
Stomach ache		124 (1.6)	1718 (2.8)	1842(2.7)
Nausea		413 (5.4)	6285 (10.3)	6698(9.8)
Vomiting		273 (3.6)	5116 (8.4)	5389(7.9)
Diarrhea		194 (2.5)	3465(5.7)	3659(5.3)
Anorexia		766(10.1)	8037(13.2)	8803(12.9)
Inflammation /lesion skin		9(0.1)	124 (0.2)	133(0.2)
Intubation		2708 (35.2)	1611(2.6)	4319(6.3)
Oxygen therapy		2203(19.8)	8949(80.2)	11152(20.0)
PCR test done		5893(76.7)	46719 (76.2)	52612(76.3)
Number of breaths per minute on Admission (breaths/min)	<5	125(2.1)	18(0.0)	143(0.3)
	5-10	109 (1.8)	85(0.2)	194(0.3)
	10-14	481 (7.9)	2610 (5.2)	3091(5.5)
	14-18	1270(21.0)	9999(20.0)	11269(20.1)
	18-22	2529(41.7)	26252 (52.5)	28781(51.3)
	22-28	1051(17.3)	7676 (15.4)	8727(15.6)
	<28	496(6.5)	3353(6.7)	3849(6.9)
Hospitalization frequency	1time	6205(80.8)	46458(75.8)	52663(76.3)
	2 time	1163(15.1)	10031(16.4)	11194(16.2)
	2 times	315(4.1)	4811(7.8)	5126(74.5)
Arterial oxygen level at the time of admission	<93%	2426 (31.6)	43814 (71.5)	46240(67.0)
	<93%	5257 (68.4)	17486 (28.5)	22743(33.0)
Hospitalization ward	Intensive Care Unit	3029(39.4)	4037(6.6)	7066(10.2)
	General	2266(29.5)	31154(50.8)	33420(48.4)
	Respiratory Isolation	2388(31.1)	26109 (42.6)	28497(41.3)
Covid-19 PCR Test results	Positive	3555(60.5)	22310(36.4)	25865(49.6)
Imaging results (X-RAY/ CT Scan)	Symptomatic	3345(95.7)	23289(93.6)	26634(93.8)

**Fig. 2:**
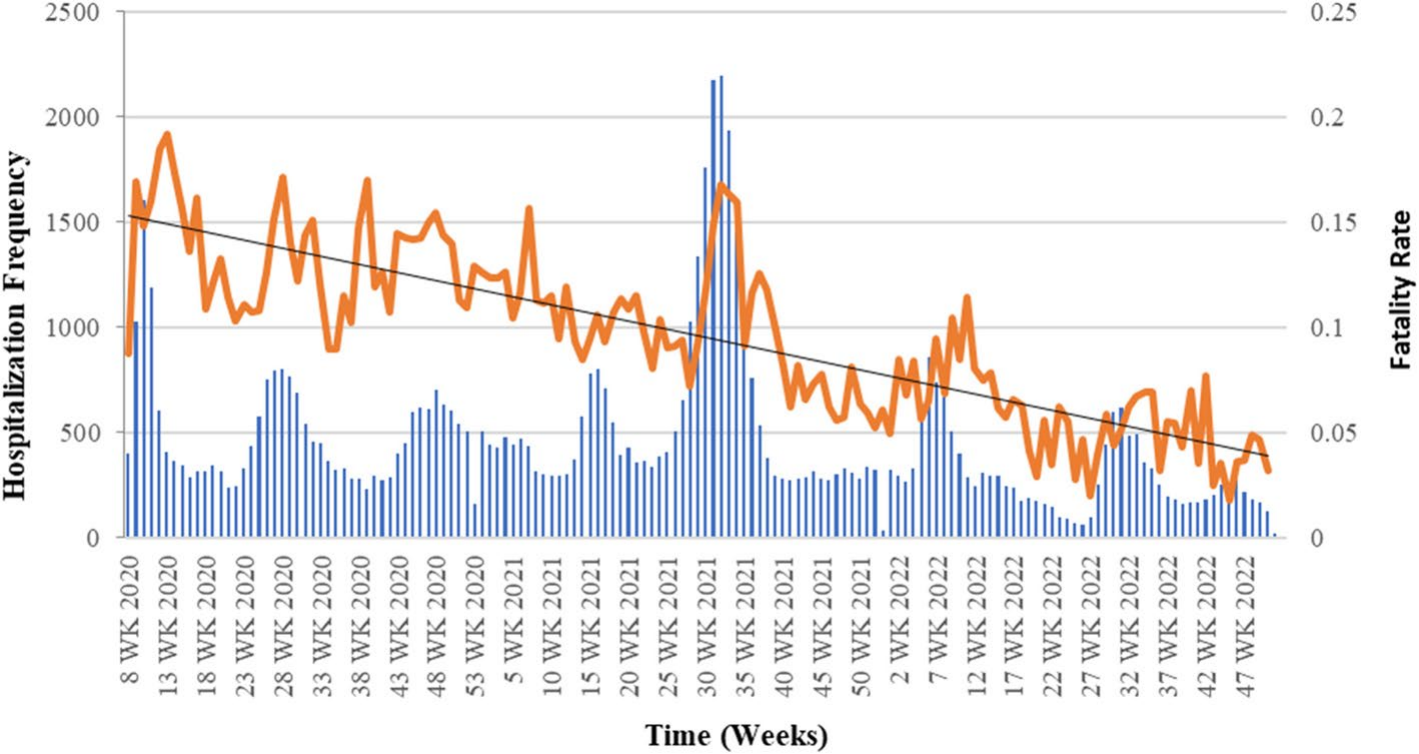
Time trend of COVID-19 hospitalization and death in Golestan provine, Iran, 2020/02/20 to 2022/12/19

The case fatality rate (CFR) of hospitalized patients with COVID-19 was 11.1% during the period under review. Also, the results show that the CFR followed a decreasing trend during this period despite significant fluctuations (Fig. 2). The mean (SD) age of deceased individuals was 63.34 (18.46) years, compared to 47.56 (24.01) years in those who survived. The highest CFR was observed in the age group above 85 years (25.9%). Death was higher in men (12.1%) than women (10.3%). The median (IQR) duration of hospital stay was 4 [5] days, and the survival probability of patients according to Kaplan–Meier analysis was 75%, 50%, and 25% on days 14, 25, and 44 of hospitalization, respectively.

**Fig. 3:**
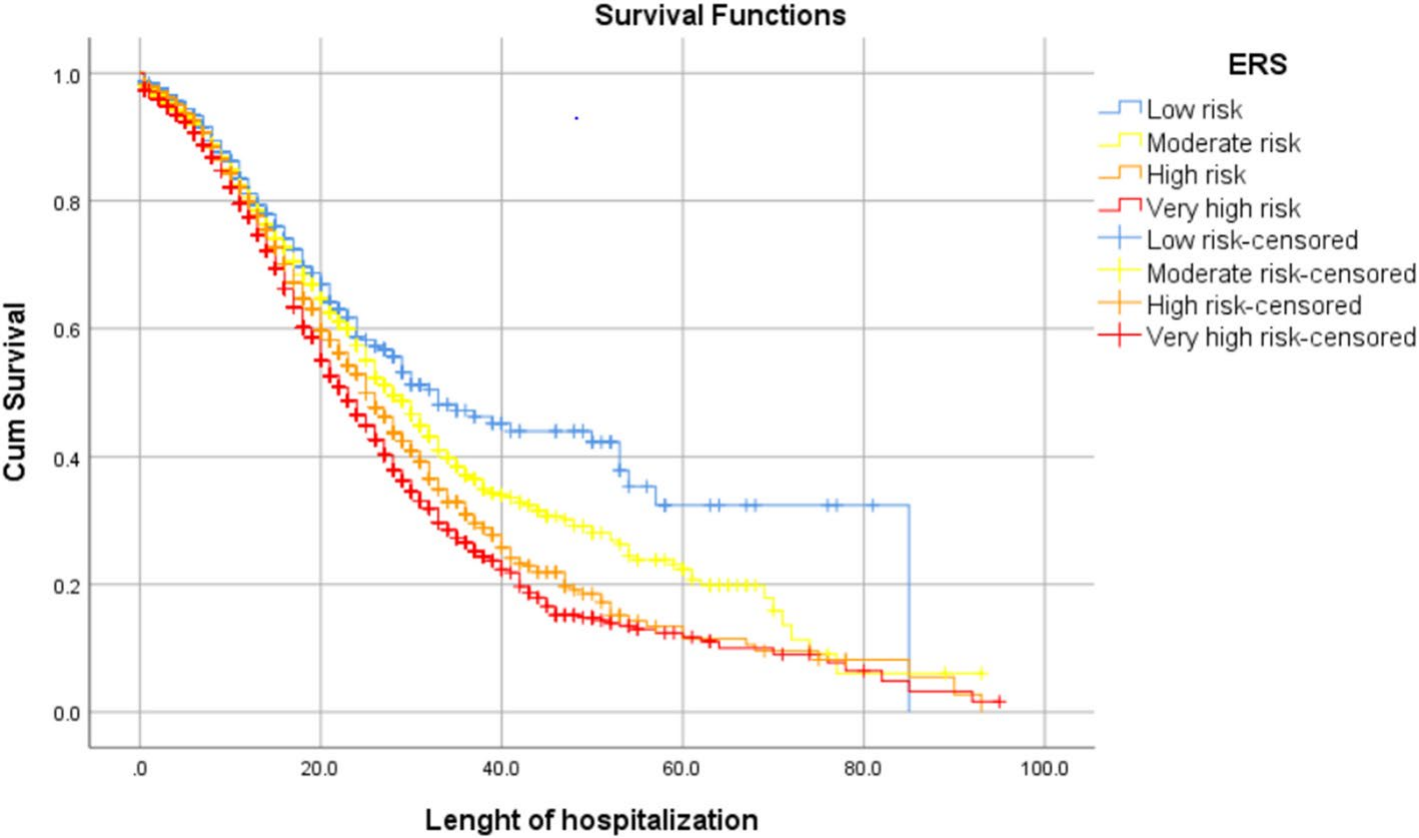
Survival rate in hospitalized patients with covid-19 based on ERS in Golestan province

The log-rank test results of factors affecting the duration of hospitalization indicate that the duration of hospitalization differed with ERS levels (Fig. 3), age groups, gender, underlying diseases, and COVID-19 severity (*P* < 0.001 for all). With increased ERS levels, COVID-19 severity, and age (except for the 18–29 and 30–39 years age groups), the duration of hospitalization decreased. The median duration of hospitalization in patients with moderate, severe, and critical COVID-19 was 50, 28, and 13 days, respectively. This duration was shorter (23 days) in those with an underlying disease than those without an underlying disease (29 days). It was 26 days in women and 25 days in men.

In the Cox survival analysis, considering the non-establishment of Cox’s proportional hazards assumption for variables such as age, gender, and disease severity classification, using Schoenfeld residuals and the extended Cox model, we considered the interaction effect of time with the mentioned variables. The raw relationship between the length of hospital stay and the ERS level showed that the risk of death at medium, high, and very high ERS levels, compared to the low-risk level, increased by 20%, 24%, and 48%, respectively (*P* < 0.001 for all). After adjusting for variables such as age, gender, city of residence, disease severity, and underlying diseases, at medium, high, and very high ERS levels compared to the low-risk level, the risk of death increased by 19%, 26%, and 56%, respectively (*P* < 0.001 for all) (Table 3).

**Table 3:** The association of Epidemic Risk Status and COVID-19 mortality using cox regression analysis

Epidemic Risk Status	Outcome		Unadjusted Hazard Ratio (95% CI )	*P*-value	Adjusted Hazard Ratio^a^ (95% CI)	*P*-value
	Death Number (%)	Discharge Number (%)				
Low risk	497 (7.7)	5993 (92.3)	Ref.	-	Ref	-
Moderate risk	2094 (10.1)	18715 (89.9)	1.20 (1.09 – 1.32)	<0.001	1.19 (1.08 – 1.31)	<0.001
High risk	1647 (10.9)	13471 (89.1)	1.24 (1.22 – 1.37)	<0.001	1.26 (1.14 – 1.40)	<0.001
Very high risk	3380(13.1)	22454 (86.9)	1.48 (1.35 – 1.63)	<0.001	1.56 (1.42 – 1.72)	<0.001

^a^Adjusted for gender, age, city of residence, disease severity classification , underlying diseases

## Discussion

The present study aimed to investigate the changes in the mortality rate in hospitalized patients with COVID-19 and delineate the relationship between the ERS level and patient survival, adjusting for individual risk factors. We also assessed patient survival in different subgroups of age, gender, presence of underlying diseases, disease severity, and ERS level. For this purpose, data recorded from the beginning of the COVID-19 pandemic to 57 months after it (68,983 individuals) in the Golestan province of Iran were used. Based on our findings, the case fatality rate (CFR) of hospitalized COVID-19 patients was 11.1%. Similarly, in other studies conducted in Iran, the CFR was 11.2–11.5% [18, 19]. In a systematic review and meta-analysis of 21 studies of hospitalized individuals with COVID-19, the CFR was 11.5% [20].

Our results showed that the adjusted risk of death was higher in moderate (19%), high-risk (26%), and very high-risk (56%) ERS levels compared to the low-risk level. It seems that one of the reasons that can lead to an increase in the death of patients is the increase in the admission rate in hospitals during peaks of the epidemic, which itself causes significant challenges for hospital management. Other studies have shown that the mortality of COVID-19 patients in hospitals is related to the increase in the hospital admission rate, with the burden on hospital capacities causing a decrease in the quality of services [12, 15]. Also, delays in visiting the hospital or hospitalization have been cited as predictors of mortality [13, 18].

In this study, the decreasing trend of CFR during the study period was noted, which is separate from the impact of the ERS level and may be due to the different variants of the viral agent or the increase in vaccination coverage. On the other hand, the treatment staff's acquisition of more experience and skill in dealing with this disease and the improvement of clinical guidelines can also be considered to decrease mortality over time [19, 20].

Our findings show that the CFR increased with the increasing age of patients. The highest CFR was observed in the age group above 85 years and the lowest in the age group 0–17 years. Most of the studies also showed that the mortality of hospitalized patients with COVID-19 increases with increasing age and decreasing immune system function [8, 9]. On the other hand, the prevalence of concomitant diseases, especially cardiovascular diseases, diabetes, and hypertension, is more common in older adults [21].

In our study, the distribution of survival time differed between men and women, and the median survival time in men who died was less than in women. Most studies also showed that the mortality rate of hospitalized patients with COVID-19 is higher in men than in women [22, 23]. A combination of behavioral, biological, social, and immunological factors may explain this difference [24, 25]. As expected, mortality was different in patients with various severities of COVID-19, with survival having an inverse relationship with disease severity. Other studies also showed that the mortality of hospitalized patients with COVID-19 increases with the severity of the disease [26, 27].

This study shows that the survival rate in people with at least one underlying disease was lower than that of people without an underlying disease (*P* < 0.001). The median survival time was 29 days in the former compared with 23 days in the latter group. In the studies conducted in Iran, compared to people without underlying diseases, patients with underlying diseases were at an increased risk of mortality in the hospital, and with the increase in the number of underlying diseases, the risk of mortality also increased significantly [28]. Having underlying diseases may be associated with a decrease in immune system function. Furthermore, these patients take more drugs, so notorious adverse drug reactions (ADRs) may also increase mortality in these patients. For example, in patients with diabetes, the normal function of the immune system is significantly impaired [29].

The study limitations include using the existing data recording system and not prospectively recording all factors affecting mortality such as viral variants and treatments. Furthermore, as the information was extracted from an online system, there is a possibility of underreporting, incorrect registration, and delay in reporting.

## Conclusion

An increment in the Epidemic Risk Status (ERS) level, while adjusting for other variables, increases the risk of death in hospitalized patients with COVID-19. Therefore, it is recommended that in the face of future epidemics and potential health events, preventive and control measures at the community level be implemented to prevent excessive patient referrals and overburdening of hospitals (such as home care, outpatient treatment centers, triage and isolation of patients based on disease risk assessment). Also, it seems that proper management of hospital capacity, including the sufficient allocation of equipment, personnel, and other resources, can reduce excessive fatigue of medical staff and improve the quality of services during the epidemic. On the other hand, the rapid and targeted increase in the reserve capacity of hospital beds (surge capacity) in the next step may have an impact on reducing patient deaths. Finally, it is recommended that in the event of a new wave of this disease, high-risk individuals, including those with underlying diseases, especially men of older ages (particularly > 85) with critical illness, be prioritized in the provision of hospital services.

## Data Availability

No datasets were generated or analysed during the current study.

## Abbreviations

COVID-19: Coronavirus disease 2019
MCMC: Medical Care Monitoring Center
ERS: Epidemic Risk Status
SD: Standard Deviation
IQR: InterQuartile Range
CFR: Case Fatality Rate
AIR: Adjusted Incidence Rate
CDC: Center for Disease Control and Prevention
